# Decoding the Attended Speaker From EEG Using Adaptive Evaluation Intervals Captures Fluctuations in Attentional Listening

**DOI:** 10.3389/fnins.2020.00603

**Published:** 2020-06-16

**Authors:** Manuela Jaeger, Bojana Mirkovic, Martin G. Bleichner, Stefan Debener

**Affiliations:** ^1^Neuropsychology Lab, Department of Psychology, University of Oldenburg, Oldenburg, Germany; ^2^Fraunhofer Institute for Digital Media Technology IDMT, Division Hearing, Speech and Audio Technology, Oldenburg, Germany; ^3^Cluster of Excellence Hearing4all, University of Oldenburg, Oldenburg, Germany; ^4^Neurophysiology of Everyday Life Lab, Department of Psychology, University of Oldenburg, Oldenburg, Germany; ^5^Research Center for Neurosensory Science, University of Oldenburg, Oldenburg, Germany

**Keywords:** EEG, AAD, speech envelope tracking, online attended speaker decoding, listening effort, selective auditory attention, attentional fluctuations

## Abstract

Listeners differ in their ability to attend to a speech stream in the presence of a competing sound. Differences in speech intelligibility in noise cannot be fully explained by the hearing ability which suggests the involvement of additional cognitive factors. A better understanding of the temporal fluctuations in the ability to pay selective auditory attention to a desired speech stream may help in explaining these variabilities. In order to better understand the temporal dynamics of selective auditory attention, we developed an online auditory attention decoding (AAD) processing pipeline based on speech envelope tracking in the electroencephalogram (EEG). Participants had to attend to one audiobook story while a second one had to be ignored. Online AAD was applied to track the attention toward the target speech signal. Individual temporal attention profiles were computed by combining an established AAD method with an adaptive staircase procedure. The individual decoding performance over time was analyzed and linked to behavioral performance as well as subjective ratings of listening effort, motivation, and fatigue. The grand average attended speaker decoding profile derived in the online experiment indicated performance above chance level. Parameters describing the individual AAD performance in each testing block indicated significant differences in decoding performance over time to be closely related to the behavioral performance in the selective listening task. Further, an exploratory analysis indicated that subjects with poor decoding performance reported higher listening effort and fatigue compared to good performers. Taken together our results show that online EEG based AAD in a complex listening situation is feasible. Adaptive attended speaker decoding profiles over time could be used as an objective measure of behavioral performance and listening effort. The developed online processing pipeline could also serve as a basis for future EEG based near real-time auditory neurofeedback systems.

## Introduction

The human auditory system enables us to follow a speaker of interest among concurrent other speakers, even in noisy environments (Cherry, 1953). Speech comprehension in a noisy listening situation relies on a listeners’ ability to segregate an auditory scene into separate auditory objects, and on the ability to attend to a relevant sound stream while suppressing irrelevant information. Paying attention to a specific sound object facilitates auditory processing and resolves competition between multiple sources (Shinn-Cunningham and Best, 2008; Bizley and Cohen, 2013). Several electroencephalographic (EEG) studies revealed robust modulations of event-related potentials by selective attention, which may act as a sensory gain-control-mechanism enhancing the responses to the attended auditory stimulus and/or downregulating the processing of the to-be-ignored stimulus (Hillyard et al., 1973; Woldorff et al., 1993; Choi et al., 2013; Jaeger et al., 2018).

Hearing impaired and normal hearing listeners differ in their performance when they have to attend to a specific speech stream presented simultaneously with competing sounds (Bronkhorst, 2000; Kidd et al., 2007; Shinn-Cunningham and Best, 2008; Ruggles and Shinn-Cunningham, 2011). These performance differences in speech intelligibility in noise cannot be easily explained by the degree of hearing loss (Peissig and Kollmeier, 1997; Gallun et al., 2013; Glyde et al., 2013) and suggest the involvement of additional cognitive factors. Listening to degraded speech may require the allocation of attentional resources to achieve successful speech comprehension (for a review see: Peelle, 2018). The resources are allocated based on task demands and the allocation is controlled by continuous performance monitoring operations to optimize speech intelligibility (Kuchinsky et al., 2016; Vaden et al., 2016). As a consequence, hearing impaired individuals following a conversation in a complex listening situation may experience higher levels of effort to achieve optimal speech comprehension and may fatigue earlier compared to normal hearing controls (Kramer et al., 2006; Holman et al., 2019; Puschmann et al., 2019).

Given the adaptive nature of attention allocation, it is likely that selective attention does not operate in a stable manner but rather fluctuates over time. This idea is supported by recent research showing that momentary attentional lapses or fluctuations in the level of attention are common (Weissman et al., 2006) and can result in erroneous behavior (Eichele et al., 2008). A time-resolved description of auditory selective attention may provide new insights into auditory processing deficits and may help to explain behavioral variabilities in complex listening situations in hearing impaired as well as normal hearing individuals. Our long-term goal is to provide this information as an auditory neurofeedback signal in near real-time, as this may serve as a basis for future auditory training applications.

Natural speech contains information on different time scales (Poeppel, 2003) and envelope modulations between 4 and 8 Hz seem to be critical for speech intelligibility (Drullman et al., 1994a, b; Ghitza, 2012). It has been found that the speech envelope of single speech streams is represented in ongoing auditory cortex activity (Luo and Poeppel, 2007; Aiken and Picton, 2008; Nourski et al., 2009; Kubanek et al., 2013) and the strength of this representation appears to be correlated with intelligibility (Ahissar et al., 2001; Doelling et al., 2014). A two competing speaker paradigm in which two spatially separated speech streams are presented simultaneously has been established by Broadbent (1952) to examine selective attention effects in a challenging listening situation with ecologically valid stimuli. Selective attention to one of two speech streams results in a stronger cortical phase-locking to the attended compared to the ignored speech envelope (Kerlin et al., 2010; Ding and Simon, 2012; Mesgarani and Chang, 2012; Horton et al., 2013; Zion Golumbic et al., 2013; Kong et al., 2014; Fiedler et al., 2019). Moreover, hearing impaired individuals show a reduced attentional modulation in cortical speech envelope tracking, which may reflect deficits in the inhibition of to be ignored signals (Petersen et al., 2017). Accordingly, monitoring the neural tracking of the to-be-attended and to-be-ignored speech stream may capture individual differences in how selective attention abilities unfold over time.

Identifying the degree and direction of attention in near real-time requires that this information can be extracted from short time intervals. Several studies have shown that attention can be reliably decoded from single-trial EEG data in the two competing speaker paradigm (Horton et al., 2014; Mirkovic et al., 2015, 2016; O’Sullivan et al., 2015; Biesmans et al., 2017; Fiedler et al., 2017; Fuglsang et al., 2017, 2020; Haghighi et al., 2017) using various auditory attention decoding (AAD) methods (for a review see: Alickovic et al., 2019). In these studies, AAD procedures demonstrated above chance-level accuracy for evaluation periods of time ranging from 2 to 60 s. In a neurofeedback application, features should be obtained as quickly as possible. This requires implementation of an online artifact attenuation procedure, as ongoing EEG data are typically contaminated by artifacts. On the other hand, this requires minimizing the evaluation interval. Current AAD procedures do not focus on adapting evaluation intervals online, which would allow the tracking of attentional fluctuations. Most studies ignore the possibility of attentional fluctuations and use a fixed evaluation interval. Yet, it is likely that attentional fluctuations influence the individual AAD accuracy and thereby contribute to performance differences which are not reflected in behavioral performance (Horton et al., 2014; Mirkovic et al., 2015, 2016; O’Sullivan et al., 2015; Puschmann et al., 2019).

Our aim was to develop a simple online AAD processing. Therefore, we implemented a single-trial decoding approach that included a fully automated online EEG artifact attenuation procedure. Individual attended speaker decoding profiles were estimated by combining the previously established AAD method with an adaptive staircase procedure, which modulated the length of the next evaluation interval based on the previous decoding outcome. The staircase served to optimize the trade-off between the duration of an evaluation interval and a participant’s individual AAD accuracy. A two competing speaker paradigm was carried out to initially validate the performance of the developed online AAD processing pipeline in a group of normal hearing listeners. By using a well-established paradigm, we expected to examine reliable effects of selective auditory attention in the normal hearing population and the derived AAD decoding performance could be compared to other AAD methods. In a first analysis the adaptive staircase procedure was evaluated offline to demonstrate that decoding performance was better than chance level. Second, the efficiency of the online EEG artifact attenuation procedure was explored by comparing the online AAD performance with AAD performance based on uncorrected EEG data. Third, parameters reflecting the attended speaker decoding performance in each testing block were analyzed and related to behavioral performance, in order to identify a possible link to the selective attention ability over time. Finally, a possible relationship between attended speaker decoding performance and subjective ratings of listening effort, motivation and fatigue was explored based on a group analysis. Listening effort is related to the speech intelligibility (determined by the speech-to-noise ratio) and typically reveals large inter-individual differences (Krueger et al., 2017).

## Materials and Methods

### Participants

Twenty one native German speaking participants between the age of 19 and 30 (mean age = 22.3; *SD* 2.7; 16 female) took part in the study. All reported no present neurological or psychiatric conditions. Audiometric thresholds of 20 dB HL or better in both ears were confirmed by pure tone audiometry at octave frequencies from 250 Hz to 8 kHz. The study was approved by the local ethics committee (University of Oldenburg, Germany) and conforms with the World Medical Association Declaration of Helsinki. All participants signed informed consent prior to the experiment and received monetary reimbursement afterward. One individual had to be excluded from the analysis due to technical problems (data loss) during the experiment, leaving a sample size of *N* = 20 for the EEG analysis.

### Task and Stimuli

To investigate if AAD based on envelope tracking is feasible in an online experiment we implemented a paradigm with two competing speakers similar to previously reported studies (Mirkovic et al., 2015; O’Sullivan et al., 2015). Participants were instructed to attend to one of two simultaneously presented speech streams throughout the entire experiment (approximately 60 min). One speech stream was presented from the right and the other from the left side to achieve a natural listening situation in which participants were able to use additional spatial cues to direct selective auditory attention. The to-be-attended speech stream and its side of presentation was not changed during the experimental session but was randomized across participants. The stimulus presentation consisted of six blocks lasting 10 min each and separated by short breaks of approximately 5 min. Before each stimulus presentation block an arrow, presented on a screen, pointed in the direction of the to-be-attended speech stream to remind participants about the attended story and its side of presentation. In the stimulus presentation blocks participants were instructed to keep their eyes open and to focus their gaze on a white fixation cross on a light gray background. During the break, subjects were asked to rate their “subjective listening effort,” “subjective motivation level,” and “subjective fatigue level.” For “subjective listening effort” participants were asked “How much effort does it require for you to follow the speaker?” (“Wie anstrengend ist es für Sie dem Sprecher zu folgen?” in German) using a categorical rating scale with seven labeled categories and six intermediate steps from “no effort” (“mühelos” in German) to “extreme effort” (“extrem anstrengend”) according to Krueger et al. (2017). “Subjective motivation level” and “subjective fatigue level” was evaluated by asking “How motivated are you now?” (“Wie motiviert sind Sie jetzt?”) and “How tired are you now?” (“Wie müde fühlen Sie sich jetzt?”). Subjective ratings of motivation and fatigue were done on the same categorical scale used for rating listening effort to achieve similar scaling between the items. After rating their subjective listening effort, motivation and fatigue level, participants were asked to fill out a multiple-choice questionnaire containing 10 questions related to the content of each speech stream in the previous block. Participants were instructed to answer as many questions as possible but were discouraged from guessing the answers to any question by choosing to leave a question unanswered if they did not know the answer. Even the questionnaire contained questions related to both speech streams, participants were further encouraged to continue attending only to the indicated speech stream and to ignore the other one.

The two speech streams consisted of fairy tales narrated in German by two professional male speakers. For each speech stream silent gaps longer than 500 ms were reduced to this length. The amplitude of both speech streams was adjusted to achieve equal loudness. A detailed description of the speech material and loudness adjustment is available in Mirkovic et al. (2016). Both speech streams were sampled at a rate of 48 kHz and presented to the participant using Psychophysics Toolbox for Matlab (Brainard, 1997), a HDSP 9632 sound card (RME, Haimhausen, Germany), a ADI 8 DS MK III DA converter (RME, Haimhausen, Germany), PA5 attenuator (Tucker-Davis Technologies, Alachua, United States, a C245BEE amplifier (NAD, Pickering, Canada) and two Sirocco S30 loudspeakers (Cambridge Audio, London, United Kingdom). The loudspeakers were located in front of the participant 45 degree to the right and to the left at ear height. The distance between loudspeaker and ear was 1.1 m. Simultaneous presentation of the two sound streams via loudspeakers resulted in a comfortable sound pressure level of 70 dB SPL, measured at the place of the participants head.

### EEG Recordings

During the experiment, participants were seated in a comfortable chair in a sound-attenuated and dimly lit booth. EEG data were collected simultaneously from two different electrode layouts, a high-density EEG cap and two cEEGrids (Debener et al., 2015) placed around each ear of the participant. The cEEGrid data will be presented elsewhere.

The high-density EEG cap consisted of 94 Ag/AgCl electrodes arranged in a customized, infracerebral electrode cap with an equidistant electrode layout (Easycap, Herrsching, Germany). Two additional electrodes were placed below the eyes to record electro-oculograms (EOG). BrainAmp amplifiers (Brainproducts GmbH, Gilching, Germany) recorded all channels against a nose-tip reference with a sampling rate of 500 Hz and band-pass filtered the data from 0.0159 to 250 Hz. Electrode impedances were kept below 20 kΩ.

### Experimental Setup

**FIGURE 1:**
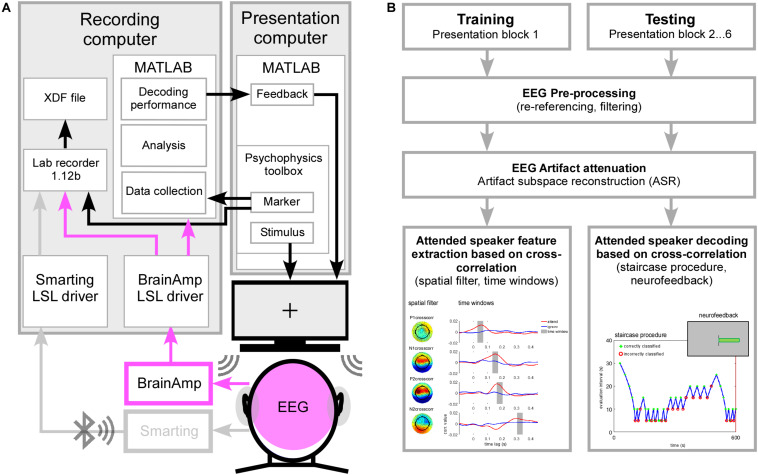
**(A)** Psychophysics toolbox was used for sound presentation and sending event markers to the Lab recorder and into Matlab on the recording computer. EEG was recorded at 96-channel high density cap (pink) and one cEEGrid attached around each ear (gray, data not shown here). High density EEG was recorded using the BrainAmp amplifiers physically connected to the recording computer. EEG data and event markers were integrated into one XDF file (extensible data format) by using Lab recorder software. High density EEG data and event marker information were collected and analyzed in Matlab. The derived attended speaker decoding performance was condensed into a feedback value, transmitted to the presentation computer and visually presented to the participant. **(B)** Schematic illustration of the training and testing procedure. EEG data derived in the training and testing phase underwent similar pre-processing and artifact attenuation. In the training procedure individual features of selective auditory attention were extracted from the cross-correlation function and related to the main positive and negative deflections (P1crosscorr, N1crosscorr, P2crosscorr, and N2crosscorr). For each deflection a spatial filter was determined and applied to the EEG data. The virtual channel time course was again cross-correlated with the attended and ignored speech envelope in order to determine a time window containing the largest attention effect. During the testing procedure spatial filters and corresponding time windows were applied to extract the attention effect based on cross-correlation values. Averaged across the main deflections a positive value indicated a correctly classified trial while a negative value indicated an incorrectly classified trial. Based on the classification outcome in the previous evaluation interval the length of the next evaluation interval was modulated in steps of 5 s to derive individual attended speaker decoding profiles. Condensed neurofeedback was presented as a visual bar at the end of each testing block.

The experimental setup is shown in Figure 1A and consisted of two personal computers connected with ethernet cable to a switch and building a small network. A presentation computer was responsible for auditory stimulus presentation, sound onset marker delivery and presentation of visual instructions and feedback information on a screen located in the booth. High density EEG cap and cEEGrids EEG signals as well as sound presentation onset markers were streamed into the network and integrated using the Lab Recorder software from the Lab Streaming Layer (LSL)^1^ package running on the recording computer. LSL enables the collection of time series from different recording modalities by handling the networking, time-synchronization and (near) real-time access to the data (Swartz Center for Computational Neuroscience and Kothe, 2015). On the same recording computer, high-density EEG data and sound presentation onset markers were additionally collected in Matlab (Version 7.14, Mathworks Inc., Natick, MA, United States) to perform the online attended speaker decoding. Using Matlab (as described in section “Online AAD Processing Pipeline”) the derived attended speaker decoding performance was condensed into a single feedback value and presented to the participant as a horizontal bar on the screen.

### Online AAD Processing Pipeline

During the experiment AAD was performed online on the high-density EEG cap data and by using customized Matlab scripts and the EEGLAB toolbox (Version 13.6.5b; Delorme and Makeig, 2004). Since selective auditory attention modulates the strength of the attended speech representation in the EEG (Kong et al., 2014; O’Sullivan et al., 2015), speech envelope tracking was realized by analyzing the EEG impulse responses to the temporal envelopes of the presented speech streams. EEG impulse responses were estimated by applying a cross-correlation analysis between EEG signals and corresponding speech envelope information.

The temporal envelopes of the clean speech were extracted following Petersen et al. (2017). After computing the absolute values of the Hilbert transform of the two speech streams the transformed signals were low-pass filtered at 15 Hz. The first order derivative was calculated to highlight prominent changes in the speech signal time course related to sound onsets of words and syllables. After half-wave rectification the resulting speech envelopes were resampled to 250 Hz. The speech envelopes of the presented speech streams were extracted offline and stored for the online EEG data analysis.

The online speech envelope tracking procedure consisted of two parts: (1) training the model needed for making attended speaker prediction was performed after the 1st presentation block on EEG data collected in that block, (2) testing the model in subsequent presentation blocks (2–6) and condensed feedback presentation at the end of each block. Note that the online, adaptive processing pipeline for AAD was fully automated and did not require any action from participants or experimenter. A schematic illustration of the training and testing procedure is shown in Figure 1B.

#### Training Procedure

After finishing the data collection of the 1st presentation block, the EEG raw data were pre-processed. This included re-referencing to common average, low pass filtering at 40 Hz (FIR filter, filter order: 100, window type: Hann), downsampling to 250 Hz and high pass filtering at 1 Hz (FIR filter, filter order: 500, window type: Hann) to remove drifts from the data. The pre-processed EEG data were submitted to a processing pipeline performing EEG artifact attenuation and deriving individual parameters for EEG based attended speaker decoding.

For online EEG artifact reduction Artifact Subspace Reconstruction (ASR) as introduced by Mullen et al. (2013) and available as EEGLAB plugin *clean_rawdata* (version 0.32) was used. ASR is based on a sliding-window Principal Component Analysis and attenuates high-variance signal components in the EEG data (for instance, eye blinks, eye movements, and motion artifacts) relative to some artifact-free calibration data reasonably well (Blum et al., 2019). To derive the required artifact-free calibration data, time windows containing abnormally high-power artifacts were automatically removed from the pre-processed EEG data by running the *clean_window* function. The function is included in the *clean_rawdata* plugin and was called based on default parameters except of the *MaxBadChannels* parameter: aiming for a very clean output we used a value of 0.075. EEG channels containing abnormal data or higher amount of line noise were identified based on inter-channel correlations by submitting the pre-processed EEG data to the *clean_channels* function (included in the *clean_rawdata* plugin). As *CorrelationThreshold* parameter a value of 0.95 was chosen meaning that EEG channels with a lower correlation value relative to the other channels were marked as abnormal. The identified bad channels were excluded from the EEG data analysis during the training and testing procedure. The obtained artifact-free calibration data were submitted to the ASR calibration method (function *asr_calibrate*) to derive a state structure containing the statistical properties of the calibration data. This state structure was submitted together with the original pre-processed EEG data to the ASR processing method (function *asr_process*). During the processing step the ASR method detects artifacts based on their deviant statistical properties and linearly reconstructs the EEG data from the retained signal subspace based on the statistical properties of the calibration data. Since ASR is processing the EEG data in chunks of 500 ms, it makes it suitable for automatic EEG artifact attenuation in online applications.

The artifact attenuated EEG data from the first presentation block were used as a training data set to derive individual parameters for the EEG based AAD. After low pass filtering at 15 Hz (FIR filter, filter order: 100, window type: Hann) the 10 min continuous training data were segmented in time periods of 30 s resulting in 20 consecutive trials. EEG impulse responses to the attended speaker stream were calculated for each channel and trial by running a cross-correlation between EEG signals and corresponding speech envelope information on time lags of −200 to 600 ms. The cross-correlation measures the similarity between EEG and speech envelope as a function of temporal displacement of one relative to the other. The derived cross-correlation coefficients range between −1 and +1. Values closer to 0 indicate no similarity, while values closer to ±1 indicate a strong linear relationship between the two signals.

The obtained EEG impulse responses at each channel were averaged across trials and revealed positive and negative deflections which resemble in their peak latencies and topographies components from the auditory evoked potential literature (Picton, 2013). From the averaged EEG impulse response we extracted the scalp distribution of the cross-correlation coefficients corresponding to the maxima and minima of the main deflections denoted P1crosscorr, N1crosscorr, P2crosscorr, and N2crosscorr based on their polarity and predefined time windows (P1crosscorr: 28–68 ms; N1crosscorr: 76–156 ms; P2crosscorr: 156–396 ms; N2crosscorr: 276–456 ms). Each of the extracted scalp distributions were used as a spatial filter in which the corresponding cross-correlation coefficients were interpreted as filter weights. Multiplying the spatial filter weights with the multi-channel EEG time course derived one virtual channel time course for each deflection (P1crosscorr, N1crosscorr, P2crosscorr, and N2crosscorr). Thereby, EEG channels with higher cross-correlation values were given more weight than those with lower values. Furthermore, EEG channels with negative cross-correlation coefficients, indicating a negative linear relationship between EEG and speech envelope time course, were reversed in phase.

Separately for each deflection, the virtual channel time course was segmented into trials of 30 s length and cross-correlated with the speech envelopes of to-be-attended and to-be-ignored speech stream. The trial averaged EEG impulse response to the to-be-ignored speaker stream was subtracted from the trial averaged EEG impulse response to the to-be-attended speaker stream to quantify the effect of selective attention on the neural tracking of speech. In the derived difference EEG impulse response the time point was determined showing the maximum positive deviation, indicating the largest attention effect. To further optimize performance of the attended speaker decoding algorithm against trial to trial variations in the EEG impulse response, an analysis time window of ±20 ms was centered on each time point. Averaging cross-correlation values across the analysis time window should increase robustness of the discriminative algorithm against random outliers.

At the end of the training procedure, the individually extracted spatial filter weights and corresponding analysis time windows at each deflection (P1crosscorr, N1crosscorr, P2crosscorr, and N2crosscorr) were stored for the attended speaker decoding performed by the testing procedure together with bad channels information and the ASR state structure necessary to run the automatic EEG artifact attenuation.

#### Testing Procedure and Condensed Feedback Presentation

EEG based AAD including automatic EEG artifact attenuation was performed by running a testing procedure at the end of each presentation block (2–6). The following processing steps were done on a single-trial level to evaluate the feasibility of online data processing. EEG raw data were pre-processed identically to the training procedure. Bad channels identified during training procedure were excluded from the data analysis and pre-processed EEG data were submitted together with the ASR state structure to the ASR processing method (function *asr_process*) to run automatic EEG artifact attenuation. The ASR state structure was updated every time it was called to account for gradual changes in the statistical properties of the EEG data over time. After performing artifact attenuation, the pre-processed EEG data were low pass filtered at 15 Hz (FIR filter, filter order: 100, window type: Hann) and submitted to the attended speaker decoding algorithm.

During AAD the extracted spatial filter weights were applied to the pre-processed multi-channel EEG time course to derive one virtual channel for each deflection (P1crosscorr, N1crosscorr, P2crosscorr, and N2crosscorr). These virtual channel time courses underwent the same processing steps as in the training procedure resulting again in EEG impulse response difference values that were then averaged across all four deflections (P1crosscorr, N1crosscorr, P2crosscorr, and N2crosscorr) and used as a decision criterion to quantify the effect of selective attention. A positive difference value indicated that the to-be-attended speech envelope was more strongly represented in the EEG compared to the to-be-ignored speech envelope and the single trial was marked as correctly classified. A negative difference value indicated a stronger representation of the to-be-ignored speech envelope in the EEG and the single trial was marked as incorrectly classified.

We used a 1-up, 1-down staircase procedure to adapt the evaluation interval (trial length) of the single trial analysis to the individual attended speaker decoding performance. Starting with an evaluation interval of 30 s for the first trial in the 2nd presentation block (i.e., 1st testing block) the evaluation interval of the following trials was varied in steps of ±5 s based on the outcome of the attended speaker decoding in the previous trial. If the current trial was classified correctly, the evaluation interval for the next trial was shortened by 5 s. An incorrect classification resulted in an extension of the next trial evaluation interval by 5 s. The lower edge of the staircase procedure was defined as an evaluation interval of 5 s, while the upper edge was not restricted. During the testing phase (2nd–6th presentation block), the staircase procedure was automatically stopped at the end of each presentation block and the value used for the first trial of the subsequent block, in order to derive a continuous attended speaker decoding profile over time.

To test the feasibility of an auditory neurofeedback application, the AAD performance reflected by the blockwise outcome of the staircase procedure was condensed into a single feedback value. The visual feedback was presented as a bar to the participant, after completing the content related questionnaires and subjective ratings of the previous presentation block. Given the infrequent presentation of the feedback value, we did not expect a benefit of the feedback on subsequent block performance and therefore did not analyze the feedback further.

### Offline Validation of the Online AAD Processing Pipeline

#### Grand Average EEG Impulse Response

In order to explore whether attention influenced the neural tracking, corresponding EEG impulse responses were extracted from the EEG data collected during the testing phase (5 testing blocks, 3000 s in total). The EEG data were pre-processed identically to the online procedure and automatic EEG artifact attenuation was applied. Impulse responses to the to-be-attended and to-be-ignored speech were calculated at each EEG channel using 30 s evaluation intervals (100 trials in total) and averaged across trials for each participant. The grand average EEG impulse response to the attended and ignored speech envelope was used to identify the main positive and negative deflections (P1crosscorr, N1crosscorr, P2crosscorr, and N2crosscorr) and corresponding topographies reflecting which electrode sites contributed most to the neural tracking of the speech envelope.

#### Grand Average Attended Speaker Decoding Profile

To derive a grand average attended speaker decoding profile the individual profiles determined by the 1-up, 1-down online staircase procedure were interpolated over the complete time course of the testing phase (5 testing blocks, 3000 s in total) in steps of 5 s and averaged across participants. A chance level attended speaker decoding profile was calculated in the offline analysis to identify at which time points the grand average profiles significantly differ from chance performance. To derive a chance level for AAD combined with the 1-up, 1-down staircase procedure we used a permutation approach. For this, individual spatial filter weights and corresponding analysis time windows extracted during the training procedure in the online phase were kept identical, while the attended speaker decoding profile was calculated offline by using the to-be-attended and to-be-ignored speech envelope from a randomly assigned part of the speech material. We repeated this procedure 10 times for each participant to derive a valid chance level decoding profile. Attended speaker decoding profiles were tested with a running Wilcoxon signed rank test across participants. The resulting p values were corrected for multiple-comparisons using the False Discovery Rate (FDR) method (Benjamini and Hochberg, 1995).

#### AAD With Mean Evaluation Intervals and Fixed Trial Lengths

For further validation of the attended speaker decoding performance, we compared the outcome of the 1-up, 1-down staircase procedure reflected in the mean evaluation interval to a traditional classification method with fixed evaluation segments. Mean evaluation intervals in each testing block were transformed to normal distribution by using inverse transformation. The transformed evaluation intervals were averaged across blocks to derive a single evaluation interval for each participant reflecting the mean performance over time. Furthermore, for each participant we calculated the accuracy of correctly classified trials offline while keeping the evaluation segment at a fixed length of 30 s. Fixed trial length decoding accuracy was correlated with the transformed mean evaluation interval by using a Pearson correlation. Across participants we hypothesized that high AAD performance on fixed 30 s intervals would be related to shorter mean evaluation intervals derived from the staircase procedure and expected a negative relationship between the variables.

#### Influence of Online EEG Artifact Attenuation on AAD Performance

A possible benefit of applying automated online EEG artifact attenuation (ASR) on AAD performance was explored by comparing the attended speaker decoding profiles against decoding profiles derived from the ASR – uncorrected EEG data. The AAD training and testing procedure was performed identically to the online processing. We expected that the implemented online EEG artifact attenuation procedure (ASR) would result in a better decoding performance, which should be reflected in shorter evaluation intervals.

### Evaluation of Behavioral Performance and Decoding Performance Parameters

#### Behavioral Performance Across Testing Blocks

After completing each testing block participants were asked to fill out a multiple-choice questionnaire containing 10 questions related to the content of each speech stream in the previous block. For each participant and testing block a sensitivity index (d’) was calculated considering the z-transformed proportion of correctly answered questions to the attended story (hits) minus the z-transformed proportion of correctly answered questions to the ignored story (false alarms). We hypothesized that differences in selective attention ability over the time course of the experiment are reflected in the sensitivity index (d’). Effects of time on behavioral performance were tested by conducting a 1 × 5 repeated measures ANOVA on the sensitivity index (d’). The factor “time” (5 levels: testing blocks 1, 2, 3, 4, 5) was defined as within-subject factor and the repeated measures ANOVA was conducted by using a general linear model. The significance level was set at *p* < 0.05. Paired-sample *t*-tests were performed as *post hoc* analyses and the False Discovery Rate method (Benjamini and Hochberg, 1995) was applied to correct for multiple comparisons.

#### Decoding Performance Across Testing Blocks

Parameters describing the individual attended speaker decoding performance in each testing block were extracted from the attended speaker decoding profile derived in the online experiment. We analyzed the mean evaluation interval and the standard deviation as descriptive parameters for the mean decoding performance and decoding fluctuation over time, respectively. Both parameters were transformed to normal distribution by using inverse transformation. We hypothesized fluctuations in the extracted parameters over the time course of the experiment. Effects of time on attended speaker decoding performance were tested by conducting 1 × 5 repeated measures ANOVAs (5 levels: testing blocks 1, 2, 3, 4, 5). Again, paired-sample *t*-tests were used to follow up effects and corrections for multiple comparisons were applied where necessary (FDR).

#### Decoding Performance and Subjective Ratings of Listening Effort

A median split based on attended speaker decoding in the EEG was used to explore a possible relationship between individual attended speaker decoding performance and subjective ratings of listening effort, motivation and fatigue. In challenging listening situations these subjective ratings may indicate differences in selective attention ability across participants even when speech comprehension is still high and does not indicate significant differences in behavioral performance. Since no explicit hypotheses could be tested this analysis was exploratory and may help to tailor future studies. Participants were divided into two groups based on their transformed mean evaluation intervals averaged across all testing blocks. The 10 participants showing the best attended speaker decoding performance in the EEG were assigned to a group of “good performers,” while the remaining 10 participants formed a group of “poor performers.” Since a non-parametric Friedman test did not indicate significant differences in the subjective ratings of listening effort [χ^2^(4) = 2.84, *p* = 0.58, *n* = 20], motivation [χ^2^(4) = 3.54, *p* = 0.47, *n* = 20] and fatigue [χ^2^(4) = 1.99, *p* = 0.74, *n* = 20] over testing blocks these values were averaged across the testing blocks and compared between performance groups by using a non-parametric Mann-Whitney *U*-test.

## Results

### Offline Validation of the Online AAD Processing Pipeline

#### Grand Average EEG Impulse Response

**FIGURE 2:**
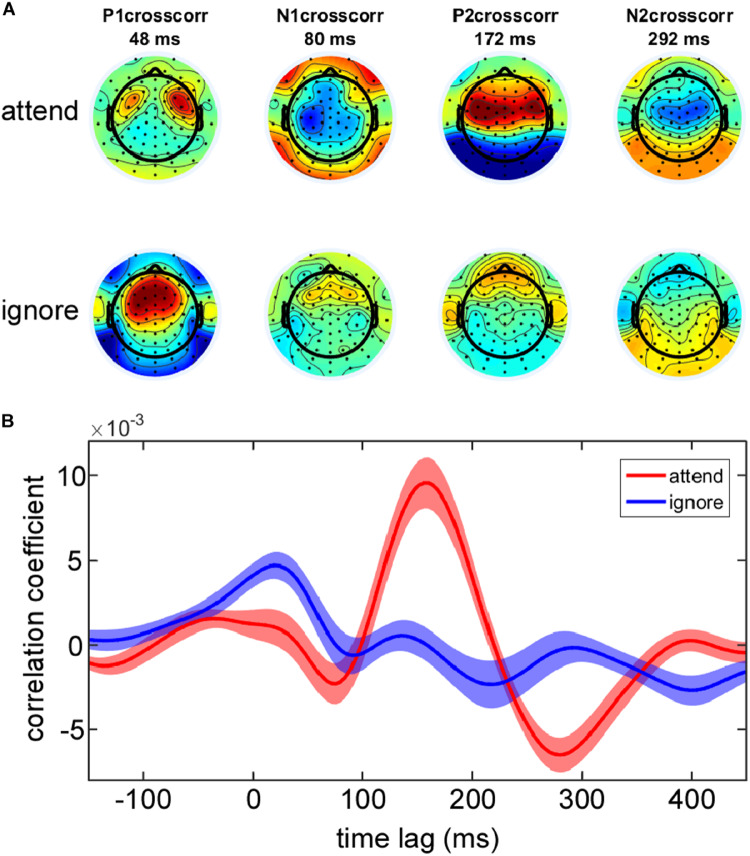
Group average EEG impulse response and corresponding topographies. **(A)** Topographies of the main positive and negative deflections (P1crosscorr, N1crosscorr, P2crosscorr, and N2crosscorr) of the group average EEG impulse response reflect which electrode sites contribute most to the neural tracking of the attended and ignored speech envelope. **(B)** EEG impulse response with ±1 SEM (shaded area) is plotted as a function of time lag separately for the attended and ignored speech envelope at EEG channel Cz.

In order to verify that attention manipulation influenced the neural tracking of the attended and ignored speech stream, corresponding EEG impulse responses were extracted from the EEG data collected during the testing phase based on 30 s intervals. In Figure 2 the grand average EEG impulse response is shown for the attended and ignored speech envelope. Based on cross-correlation we found robust responses to the attended speech envelope with peaks in correlation values at time lag 48, 80, 172, and 292 ms corresponding to the P1crosscorr, N1crosscorr, P2crosscorr and N2crosscorr components from recent speech envelope tracking literature (Horton et al., 2013; Kong et al., 2014; Petersen et al., 2017; Mirkovic et al., 2019). The scalp distributions at the peak latency correlations to the attended speech envelope showed bilateral foci over temporal and frontal electrode sites (Figure 2A). Inspection of the EEG impulse response (Figure 2B) to the ignored speech envelope suggested that selective attention had a major impact on phase-locking to the ignored speaker stream. While the EEG impulse response to the ignored speech envelope showed a clear positive peak at 48 ms corresponding to the P1crosscorr, all other subsequent deflections were strongly reduced in amplitude, possibly due to a suppression of the to-be-ignored speaker. In accordance with Kong et al. (2014) we found a stronger P1crosscorr amplitude in the EEG impulse response to the ignored speech envelope compared to the attended envelope, which was even reflected in time lags before 0 ms.

#### Grand Average Attended Speaker Decoding Profile

**FIGURE 3:**
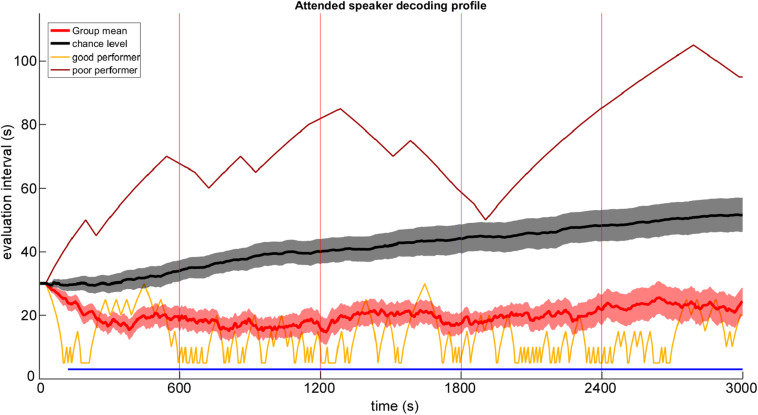
Group mean attended speaker decoding profile (red line) with ±1 SEM (red shaded area) plotted as a function of time. Single subject results are given for the worst and best performing participant. Black line and gray shaded area indicates the chance level with ±1 SEM determined by the permutation approach. The horizontal blue line marks a significant difference between attended speaker decoding profiles and chance level (running Wilcoxon signed rank test, *p* < 0.05, False Discovery Rate corrected). Red vertical lines mark pause intervals between testing blocks.

The group averaged online attended speaker decoding profile, which was derived by an online AAD processing pipeline combined with a staircase procedure is shown in Figure 3 (red line) over the entire testing phase of 3000 s. Descriptively, during the first testing block a decrease in evaluation interval was visible while from the second testing block on, a modest increase in the group-mean evaluation interval was observable. The estimated chance level decoding performance is represented as a black line, which showed a gradual increase in the length of the evaluation interval across testing blocks over time as well. A running Wilcoxon signed rank test revealed a significant difference between chance level and attended speaker decoding performance from 125 s on and persisted throughout the remaining testing phase. Notably, attended speaker decoding performance differed strongly across participants, as illustrated by showing two individual profiles representing the best (yellow line) and worst (brown line) performance.

#### AAD With Mean Evaluation Intervals and Fixed Trial Lengths

**FIGURE 4:**
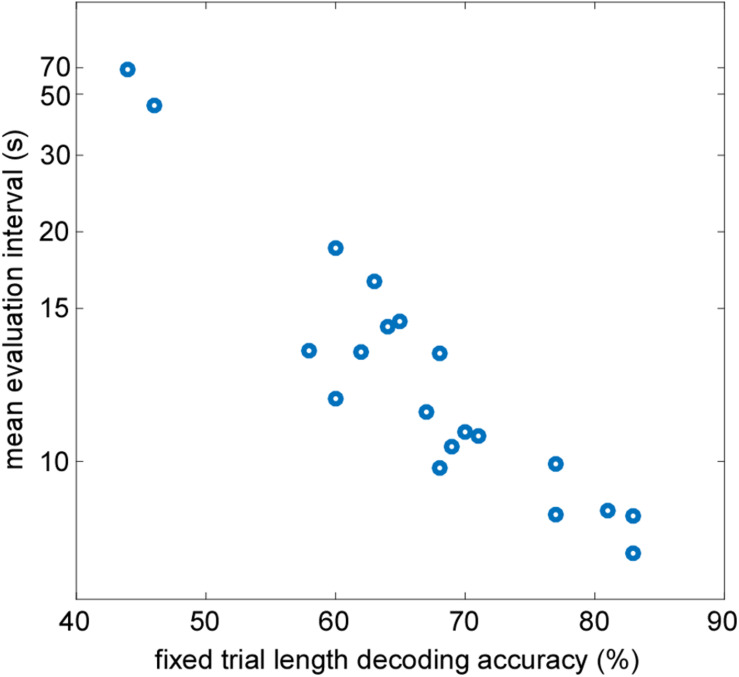
Correlation between individual mean evaluation intervals determined by the staircase procedure and attended speaker decoding accuracy calculated at fixed evaluation intervals of 30 s (r_Pearson = -0.93, *p* < 0.001).

To prove the validity of the developed AAD processing pipeline further, we compared the outcome of the adaptive staircase procedure reflected in the mean evaluation interval to a traditional classification method with fixed evaluation segments. It was expected that both methods should provide comparable decoding performance on an individual level. Averaged across participants the mean length of the evaluation interval determined by the staircase procedure was 12.2 s (range 8.3–67.6 s). The offline analysis using a fixed trial length of 30 s resulted in a group mean decoding accuracy of 67% (range 44–83%). In Figure 4, individual mean evaluation intervals are plotted as a function of individual fixed trial length decoding accuracy. A Pearson correlation revealed a strong negative linear relationship between the variables [*r*(18) = −0.93, *p*< 0.001]. In other words, participants with high fixed trial length decoding accuracy reached smaller mean evaluation intervals determined by the staircase procedure.

#### Influence of Online EEG Artifact Attenuation on AAD Performance

**FIGURE 5:**
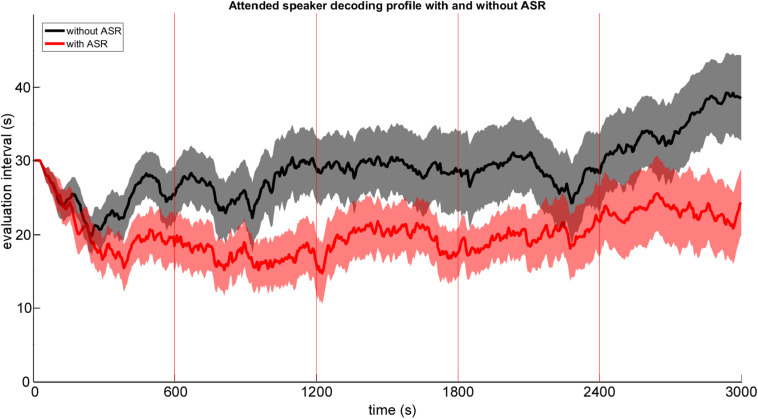
Group mean attended speaker decoding profile with ±1 SEM (shaded area) plotted as a function of time derived from ASR artifact attenuated EEG data (red) and ASR uncorrected EEG data (black).

A possible benefit of applying automated online EEG artifact attenuation (ASR) on AAD performance was explored by comparing the attended speaker decoding profiles against decoding profiles derived from the ASR – uncorrected EEG data. In Figure 5 the grand average attended speaker decoding profile is shown for corrected (with ASR – red) and uncorrected (without ASR – black) EEG data. As expected, we found that the implemented online EEG artifact attenuation procedure resulted in a better decoding performance. On a descriptive level this performance benefit is reflected in 5–10 s shorter evaluation intervals derived by the adaptive staircase procedure. Especially, in the last testing block the EEG artifact attenuation seemed to outperform the uncorrected processing. A possible explanation may be that participants fatigued earlier toward the end of the experimental duration which is often accompanied by an increase in EEG artifact (i.e., eye blinks and movements) occurrence.

### Evaluation of Behavioral Performance and Decoding Performance Parameters

#### Behavioral Performance Across Testing Blocks

**FIGURE 6:**
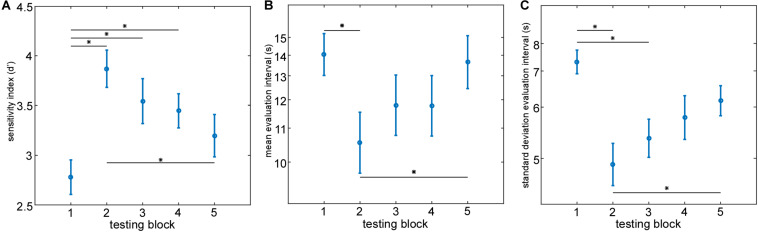
Evaluation of individual behavioral performance and attended speaker decoding performance parameters. Group mean behavioral sensitivity index (d’) **(A)**, group mean evaluation intervals **(B)** and group standard deviation evaluation interval **(C)** are plotted as a function of time (testing block). Asterisks mark a significant difference between testing blocks (paired-sample *t-*test, *p* < 0.05, False Discovery Rate corrected). Error bars represent ±1 SEM.

On the behavioral level the analysis of the questionnaire data revealed that all participants followed the instructions by attending to the indicated speech stream. On average, participants correctly answered 81.25% of the questions on content presented in the to-be-attended speech stream and only 1% of questions on the to-be-ignored speech stream. For each participant and testing block a sensitivity index (d’) based on the content related questionnaire was calculated to identify differences in behavioral performance across testing blocks (Figure 6A). On a descriptive level, the mean behavioral performance increased from testing block 1–2, as reflected in a steep increase in the sensitivity index (d’), and gradually decreased over time from testing block 2–5. Effects of time on behavioral performance were tested by conducting a 1 × 5 repeated measures ANOVA on the sensitivity index (d’) across testing blocks. The 1 × 5 repeated measures ANOVA revealed a significant main effect of time [*F*(4, 19) = 7.06, *p*< 0.0001]. *Post hoc* comparisons using two-tailed paired-sample *t-*tests identified a significant difference in the sensitivity index (d’) between testing blocks 1 and 2 [*t*(19) = −5.49, *p* < 0.001], testing blocks 1 and 3 [*t*(19) = −4.59, *p* < 0.01] as well as between testing blocks 1 and 4 [*t*(19) = −2.72, *p* < 0.05] while a significant decrease in behavioral performance from testing blocks 2–5 was evident [*t*(19) = 3.96, *p* < 0.01].

#### Decoding Performance Across Testing Blocks

Based on the online attended speaker decoding profiles we extracted the mean evaluation interval and the standard deviation evaluation interval in each testing block as descriptive parameters reflecting the individual decoding performance and its variation over time. We hypothesized those differences in selective attention ability over the time course of the experiment to be reflected in the extracted descriptive parameters. Separate repeated measures ANOVA’s were conducted on the mean evaluation intervals (Figure 6B) and its standard deviation (Figure 6C). Descriptively, both parameters showed a similar behavior over testing blocks. The best attended speaker decoding performance was achieved in testing block 2 reflected in the smallest mean evaluation interval and standard deviation. From testing block 2–5 a gradual increase in mean evaluation interval and standard deviation was apparent. The 1 × 5 repeated measures ANOVA on transformed mean evaluation intervals with the factor ‘time’ revealed a significant main effect of time [*F*(4, 19) = 4.62, *p*< 0.01]. *Post hoc* comparisons using two-tailed paired-sample *t* tests identified a significant decrease in mean evaluation interval from testing blocks 1–2 [*t*(19) = 3.37, *p* < 0.05] and a significant increase in mean evaluation interval from testing blocks 2–5 [*t*(19) = −3.74, *p* < 0.05]. A similar behavior in the time course was visible for the standard deviation of the evaluation intervals across testing blocks. Here, the 1 × 5 repeated measures ANOVA revealed a significant main effect of time [*F*(4, 19) = 6.39, *p*< 0.001] too. *Post hoc* comparisons using two-tailed paired-sample *t*-tests identified a significant decrease in the standard deviation from testing blocks 1–2 [*t*(19) = 4.42, *p* < 0.01] as well as from testing blocks 1–3 [*t*(19) = 3.86, *p* < 0.01] while a significant increase in standard deviation from testing blocks 2–5 was evident [*t*(19) = −3.14, *p* < 0.05].

#### Attended Speaker Decoding Performance and Subjective Ratings of Listening Effort

**FIGURE 7:**
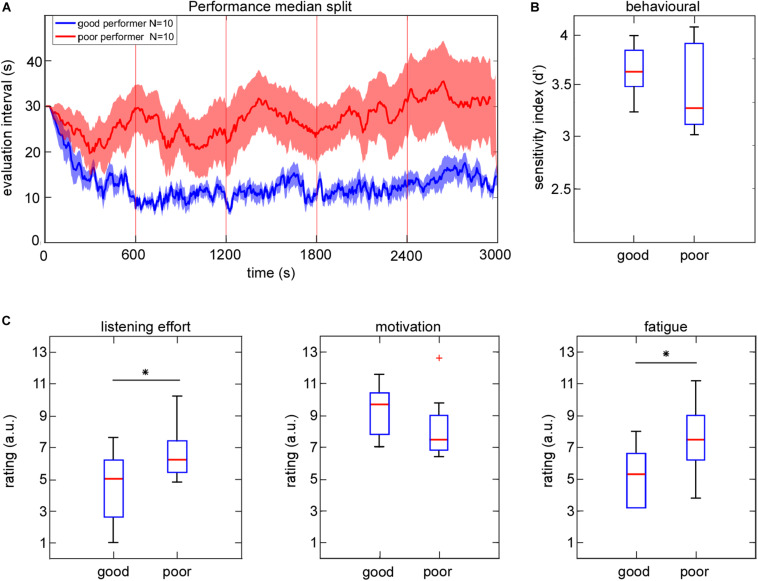
Group median split based on attended speaker decoding performance averaged across testing blocks. **(A)** Group means in performance are plotted as a function of time. The shaded areas represent ±1 SEM. **(B)** Box plot contains the behavioral sensitivity index for good and poor performer. **(C)** Box plots contain subjective ratings of listening effort, motivation and fatigue averaged across all testing blocks for the good and poor performer. Asterisks mark a significant difference in mean subjective ratings between groups (Mann–Whitney *U*-test, *p* < 0.05).

A possible relationship between individual attended speaker decoding performance and subjective ratings of listening effort, motivation and fatigue was explored by using a group median split based on the mean evaluation intervals. Figure 7 shows the good and poor performers separately for the group averaged attended speaker decoding profile (Figure 7A), behavioral performance reflected in the sensitivity index (Figure 7B) and subjective ratings of listening effort, motivation and fatigue (Figure 7C). On a descriptive level, the groups differed strongly in the group averaged attended speaker decoding profile over time. While good performers showed more stable performance in their mean evaluation interval over testing blocks the poor performers’ averaged profile indicated more pronounced fluctuations over time. Note that the group differences in attended speaker decoding performance were not reflected by differences in behavioral performance (*Z* = 0.95, *p* = 0.34). Interestingly, good and poor performers differed significantly in their subjective ratings of listening effort and fatigue. The group of good performers reported on average less listening effort (*Z* = −2.04, *p* < 0.05) and less fatigue (*Z* = −2.2, *p* < 0.05) compared to the group of poor performers. Subjective ratings of motivation were descriptively higher in the group of good performers but failed to reach significance (*Z* = 1.71, *p* = 0.09).

## Discussion

In this study, we developed an online processing pipeline performing AAD on short segments of EEG data to detect the direction and level of attention in a two competing speaker paradigm. The implemented AAD method was combined with an adaptive 1-up, 1-down staircase procedure in order to optimize the trade-off between the duration of evaluation interval and the individual decoding performance. The developed AAD processing pipeline was applied in an online experiment to capture individual attended speaker decoding profiles over time. We hypothesized that exploring these profiles may provide new insights into fluctuations of selective attention and its relation to behavioral performance.

The offline analysis confirmed that the implemented AAD method, which was based on EEG impulse responses to the speech envelope, captured selective auditory attention effects, as reported previously (Horton et al., 2013; Kong et al., 2014; Petersen et al., 2017; Mirkovic et al., 2019). Offline validation of the adaptive procedure revealed a robust relationship between the mean evaluation intervals derived by the staircase method and attended speaker decoding accuracy determined by a classical fixed trial length decoding approach. The implemented online EEG artifact attenuation procedure (ASR) had a beneficial effect on the attended speaker decoding performance resulting in 5–10 s shorter evaluation intervals compared to the artifact uncorrected EEG data, suggesting that the online processing pipeline was functioning reasonably well. This interpretation is also supported by our analysis of the attended speaker decoding profiles over time. For each participant and testing block we extracted the mean evaluation interval and its standard deviation from the individual attended speaker decoding profiles. On the group level the mean evaluation interval and the standard deviation across testing blocks was closely related to the behavioral performance (d’). Here, shorter mean evaluation intervals and fewer fluctuations in the profile were related to better behavioral performance. Additionally, an exploratory analysis between groups indicated that individuals with poorer attended speaker decoding performance experienced higher listening effort and fatigue over the time course of the experiment. In the following, the benefits and limitations of these procedures will be discussed.

Adaptive staircase procedures are frequently used to determine performance levels (Levitt and Rabiner, 1967). An adaptive 1-up, 1-down procedure is well suited to target a performance level of 50% correct responses, i.e., reveals a detection threshold. This procedure requires careful consideration, since 50% decoding accuracy in a two-class classification problem reflects chance-performance. To exclude the possibility that the captured attended speaker decoding reflected random decision profiles, we estimated the chance-level based on permutation tests (Ojala and Garriga, 2010; Combrisson and Jerbi, 2015). These analyses revealed an increase in the mean chance-level evaluation interval over time. This increase can be explained by the definition of the staircase procedure which was restricted to a minimum length of 5 s for the evaluation interval, but the maximum evaluation interval was not explicitly limited. Hence, for individuals performing at chance-level the evaluation interval would at best fluctuate around the initial evaluation interval of 30 s, or even increase over time, as observed in the permutation tests. Most individuals, however, improved at least initially from the first to the second testing block, suggesting that the attended speaker decoding profiles were not driven by stimulus properties and rather reflected individual profiles of attentive listening.

Looking at the overall decoding performance of the AAD processing pipeline a fixed trial length interval of 30 s revealed in the offline analysis a group average decoding accuracy of 67%. This accuracy level is considerably lower compared to other studies using linear spatio-temporal decoders, which reach decoding accuracies of around 90% (Mirkovic et al., 2015; O’Sullivan et al., 2015; Das et al., 2016). Two reasons may explain this performance difference. First, our processing pipeline followed a strictly chronological approach, that is, we used only the first 10 min of EEG data as a training set to derive individual features of selective attention. More common cross-fold validation strategies may outperform a chronological approach, as they use much more data for classifier training (commonly 90% of all data) and, due to cross-fold sampling, thereby compensate for non-stationarities. However, for online applications these options do not apply, and therefore a chronological processing strategy provides a more realistic result.

A second aspect explaining differences in decoding performance might be the implemented decoding procedure. State-of-the art attended speaker decoding methods focus on optimizing multivariate linear regression models to estimate the speech envelope of the attended speech stream from the EEG data (Mirkovic et al., 2015; O’Sullivan et al., 2015; Das et al., 2016, 2018; Biesmans et al., 2017; Fuglsang et al., 2017), or use deep learning procedures (de Taillez et al., 2017; Ciccarelli et al., 2019). In these studies, the process of model estimation and optimization can be computationally heavy and is therefore not well suited for the near real-time application. Recently, a framework aiming for real-time AAD based on sparse adaptive filtering was proposed by Miran et al. (2018) showing promising results. However, in most online procedures there is a trade-off between algorithm complexity and computation time. As our focus was on a near real-time application, we have opted for a straightforward and low complexity procedure. The chosen cross-correlation approach fitted to these requirements but indicated some temporal smearing of the EEG impulse response, which was visible in the P1crosscorr to the ignored speech envelope. This temporal smearing is caused by the low frequency characteristic of the speech envelope which maps to the EEG signal at overlapping time lags (Crosse et al., 2016). In our study, individual spatial and temporal information of selective attention effects were accounted for by extracting spatial filters and analysis time windows at specific deflections of the EEG impulse response to the speech envelope. The chosen methods condensed features of selective attention across EEG channels and relevant time windows and thereby allowed for a computationally inexpensive attended speaker decoding.

Offline evaluation included a correlation analysis to explore whether the mean online evaluation intervals extracted from the temporal attention profiles are linked to classical decoding accuracy calculated based on fixed trial length intervals of 30 s. A strong correlation revealed that the mean evaluation interval derived by the staircase procedure captured individual decoding performance in the same way as the classical decoding accuracy, while not relying on fixed evaluation intervals. Hence, while the adaptive online procedure does not miss stable individual differences as typically revealed by offline analysis (Choi et al., 2014; Bharadwaj et al., 2015; Puschmann et al., 2019) it optimizes the time interval necessary for attended speaker decoding and thereby reveals attentive listening profiles.

Individual differences in EEG based AAD performance in competing speaker scenarios have been observed before (Horton et al., 2014; Mirkovic et al., 2015, 2016; O’Sullivan et al., 2015). We hypothesized that these decoding performance differences might be related to the individual selective attention ability over time. To reveal more robust interpretations, differences in the attended speaker decoding profiles over time were related to the behavioral performance. For each participant and testing block we extracted the mean evaluation interval and its standard deviation from the attended speaker decoding profile as well as the behavioral sensitivity index (d’). Our statistical analysis revealed that attended speaker decoding performance changed significantly over the time course of the experiment. Best decoding performance was evident in the second testing block followed by a gradual decrease in decoding performance until the fifth testing block. This decrease in decoding performance was characterized by longer mean evaluation intervals necessary for AAD and a higher standard deviation indicating larger fluctuations in the staircase performance. A possible explanation might be that attentional lapses or fluctuations in the level of attention occurred more often toward the end of the experiment as a result of the monotonic and demanding selective listening task. Our behavioral data analysis supports this idea by revealing a similar performance pattern over testing blocks closely following the attended speaker decoding performance. A possible link between decoding performance and behavioral performance is further supported by recent research showing a clear relationship between the speech envelope tracking in the neural data and intelligibility scores to the attended speech signal (Ding and Simon, 2013; Doelling et al., 2014). Taken together, our method seems to be able to evaluate fluctuations in the level of attention, as it is based on an objective measure of attended speaker processing.

As an additional finding, our exploratory analysis indicated that poor attended speaker decoding individuals reported significantly higher listening effort and fatigue when compared to good performing individuals. Note that a group difference in behavioral performance was not evident. This result is in line with research proposing that an increase in cognitive resources (such as working memory) may help to compensate for individual selective attention modulation deficits (Shinn-Cunningham and Best, 2008). Over short periods of time, investment of additional cognitive resources may help to enhance intelligibility. Unfortunately, we did not find significant differences in subjective ratings of listening effort, motivation and fatigue over the time course of the experiment in line with the found behavioral effects. In contrast, the study of Krueger et al. (2017) revealed a clear link between the speech-to-noise ratio determining the speech intelligibility and subjective ratings of listening effort. These differences in outcome may be explained by our study design in which the speech-to-noise ratio was not explicitly modulated but was rather stable over the time course of the experiment. Indeed, good and poor performers differed in their subjective ratings of listening effort and fatigue already from the first testing block on, which was supported by the group difference in their attended speaker decoding profile. We propose that evaluating the selective attention effects between the attended and ignored speech envelope over time in the EEG could potentially serve as an objective measure of listening effort. While theoretical underpinnings and the clinical meaning of listening effort seem poorly developed, an objective measure of listening effort and listening related fatigue would be valuable and complement the variety of methodologies including self-report, behavioral and physiological measures (McGarrigle et al., 2014). Especially, in hearing impaired individuals our procedure may help to capture intra- and inter-individual differences and could be useful in evaluating assistive listening devices.

In summary, our results are consistent with other studies showing that normal-hearing listeners vary widely in their selective attention abilities (Ruggles and Shinn-Cunningham, 2011; Choi et al., 2014). Individual differences in selective auditory attention may be directly related to efficacy of executive cortical processes (Choi et al., 2014) and could be related to differences in subcortical encoding of relevant temporal features necessary for auditory object formation (Ruggles et al., 2011; Bharadwaj et al., 2014). While our analysis does not allow conclusions about the factors contributing to individual differences in temporal auditory attention profiles, we offer an efficient EEG processing pipeline that can help to capture how selective auditory attention fluctuates in complex listening scenarios over time. In a future application, our online AAD processing pipeline could serve as a basis for the development of an auditory neurofeedback training system. Providing information about selective attention fluctuations in near real-time to the participant may help to improve individual listening performance.

## Data Availability Statement

The datasets generated for this study are available on request to the corresponding author.

## Ethics Statement

The studies involving human participants were reviewed and approved by the Kommission für Forschungsfolgenabschätzung und Ethik, University of Oldenburg, Oldenburg, Germany. The patients/participants provided their written informed consent to participate in this study.

## Author Contributions

MJ performed data acquisition. MJ analyzed the data and wrote the manuscript to which BM, MB, and SD contributed with critical revisions. All authors designed the experiment, approved the final version, and agreed to be accountable for this work.

## Conflict of Interest

The authors declare that the research was conducted in the absence of any commercial or financial relationships that could be construed as a potential conflict of interest.

## Footnotes

1 https://github.com/sccn/labstreaminglayer
